# Ten simple rules for researchers while in isolation from a pandemic

**DOI:** 10.1371/journal.pcbi.1007946

**Published:** 2020-06-25

**Authors:** Hoe-Han Goh, Philip E. Bourne

**Affiliations:** 1 Institute of Systems Biology, Universiti Kebangsaan Malaysia, UKM Bangi, Selangor, Malaysia; 2 School of Data Science, University of Virginia, Charlottesville, Virginia, United States of America; Dassault Systemes BIOVIA, UNITED STATES

## Introduction

The scale and intensity of the coronavirus disease 2019 (COVID-19) worldwide pandemic is unprecedented in all our lifetimes. It has changed our lifestyles and our workstyles, in a manner and to a degree that is likely to persist for some time. Here we offer some guidance, in the familiar ten simple rules format, for how to navigate a stressful situation, considering it realistically as both a curse and an opportunity. This is written for all of us involved in scientific research—graduate student, postdoc, academic, staff scientist, in academia, government or industry. Each such person has so much to contribute in a time of need but is simultaneously also a member of a worldwide population under threat. What can one do in a time like this?

## Rule 0: Take care of yourself

This is so fundamental that we have taken the liberty of calling it Rule 0. As one reviewer put it, “put on your own oxygen mask before taking care of others.” What use are you to your loved ones and our society at large if your mental and physical state are less than optimum, or worse still, if you do not survive?

## Rule 1: Believe in your own science and influence views of others

You may be a researcher who studies the basic biochemistry of infectious diseases or who performs pandemic modeling—we need all your dedication and expertise as we collectively work through this threat. More likely, your scientific training is in a variety of other fields. Use that training. Review the literature and the public data being produced, understand, and explain to others the need for seemingly onerous measures such as social distancing; you can use past pandemics as your guide [1]. Take every opportunity to use your scientific understanding to explain the importance of protective clothing, isolation, and appropriate hygiene. It can be read anywhere but so can false or misleading claims. Use your scientific knowledge to influence others as best you can with facts and solid arguments, never shying away from clearly indicating what we do not know (that’s just as much a part of science as what we do know). Science—seriously undervalued in many parts of the world in recent years—has almost literally overnight become what the general population craves to understand, so as to grasp their predicament. Use your training and knowledge wisely and effectively, allowing all to benefit. Do so via social media, such as Facebook, Twitter, Instagram, and LinkedIn, to disseminate useful information to the general public and reinforce #StayAtHome.

## Rule 2: Show leadership in goal setting and understanding

You do not need to be a leader to show leadership. We can all lead in our own way. Whether it is as a group leader providing strength, comfort, compassion, flexibility, and direction to your team or as a graduate student providing the same to fellow graduate students or undergraduates, it all counts. It counts for compassion shown to those less fortunate. Researchers are one of the least likely groups to be seriously disadvantaged. Remember and act on that. Leadership comes in many forms, but one that might help here is the setting of new goals in times of unsettled circumstances. Labs and individuals have goals. Working towards those goals in uncertain times provides a sense of purpose and accomplishment, which would seem so important. Goals can be in various time spans, from daily to the time span of the pandemic and beyond (months, years). Goal setting is particularly difficult for experimental work when the lab is closed and equally difficult for young researchers when a thesis or paper needs to be completed. Try and be creative with goals that are achievable and that offset the sense of loss. Another form of leadership is to appropriately complement those that support your research, in whatever way. This is important at the best of times, but particularly so at the worst of times.

Show leadership in understanding. As researchers, for all that is lost, it is mostly trite relative to what many have lost by way of family members, jobs, and a way of life. Look to lead efforts that address this imbalance either through university or other programs that reach out to the community. You have skills that can help others—employ them. If you can’t find ways, then at least do more for your profession—tutor others remotely, review more, etc.

## Rule 3: Follow institutional guidance and provide feedback

By institution, we mean everything from the government (federal, state, and local) to your workplace to your individual laboratory. Enormous effort has likely gone into contingency planning, albeit not exactly for the scenario that we now face. Follow those plans, even if they have been made late relative to the spread of the virus. Those plans include how best to work remotely and knowing your classification tier (your level of “essentiality” within your organization—are you central or not to the operational capacity and wellbeing of the organization?). Essentiality can relate to the care and maintenance of laboratory animals, equipment, or materials for which safety is paramount (e.g., radioisotopes), your students, etc. Act according to that essentiality. Each of us occupies a unique niche. If a Contingency of Operation Plan (COOP), guideline, or something similar exists, study it and follow directives from your organization, as your safety and wellbeing are their top priority. (If you’re unsure if one exists, ask your immediate supervisor.) Be aware of support groups and other mechanisms to help you through these difficult times. Finally, provide feedback on the guidance you are receiving, good or bad—providing such guidance is part of an agile process. This is particularly important when the plan is not clear or does not help you as a stakeholder. Slack channels are a good option for you to try and get involved in contingency planning.

## Rule 4: Embrace a new work habit and environment

How science is conducted has changed, almost overnight. Some of what you did before will likely not be possible, but as you will see in the rules that follow, new opportunities arise. Likely, most of your computational work can continue—essential staff (Rule 3) will keep servers, high-performance computing facilities, networks, etc. running, albeit under greater strain than usual. Compensate for the loss of other forms of work and try and recreate yet others as best you can while being remote. Set up your home office to be comfortable and functional if it is not already. You will have likely gained time that was previously spent on commuting; use that time wisely. Embrace the new opportunities that exist but also consider the physical and mental wellbeing that comes from keeping a regular schedule and associated calendar, diet, exercise regime, etc.

## Rule 5: Virtual communication is the new normal—Maintain a team spirit

We are social animals in both personal and professional lives. Most of us have the technology to create some level of virtual normality in our otherwise physical communications. Use all the synchronous and asynchronous tools you have at your disposal on both computer and cellular networks to recreate as much as possible of the normal. You can still have your regular group meetings virtually via Zoom, Google Meet, Microsoft Teams, etc. Do turn on your camera during video calls as seeing familiar faces helps to alleviate the sense of loneliness. It also forces you to dress accordingly and keeping an organized workspace as a reminder that you are still working in your cozy home. Keep the team spirit going with regular communications and checking in with your colleagues if they are coping well or need assistance. Slack channels are good for this; physical presence is certainly irreplaceable, but a silver lining is that we will be in a better place when the pandemic subsides because of the extreme stress-testing of technology. To illustrate this point from our own experience, we have put classes online that would have taken years to achieve otherwise. Our faculty have quickly embraced technologies they would otherwise ignore; we have records of lab meetings, lectures, workshops, etc. that we would not otherwise have. In short, our scientific digital footprint has expanded significantly out of necessity; the most advantageous and beneficial of our new habits will persist in our post-pandemic world. Recognize this and prepare for it—do not be shy or reticent on the new format and opportunities to communicate your science.

## Rule 6: Look for new ways to contribute

It is amazing and extremely heartening to see scientists come together in the face of adversity. We all have a part to play in the face of this pandemic—to return us to a sense of normality and to make sure that, as a society, we learn from these tragic events. Although open science (sharing knowledge, data, software, etc.) should be the norm always, it has become much more imperative now. Use this opportunity to both give and take. Make your data, software, and papers immediately available through public resources and preprint servers. Donate anything from your lab that is not being used when it may benefit others. You can seize this opportunity to develop online courses [2] or workshops [3] based on the various open learning platforms available nowadays. This is not only useful to share your knowledge and skills but also helps build your scientific reputation, even in times of adversity. This is especially true for data scientists in teaching various bioinformatics and programming skills. It is important to keep up to date with the latest development of the pandemic situation. What can you contribute both to society and to science, as a researcher helping combat the pandemic? It could demand more than “thinking outside of the box” to throw away the box in helping those at the front line!

Remember researchers are disadvantaged in different ways. The more experimental your work, the more you are likely disadvantaged. Do what you can for the most disadvantaged. Continue to support scientific publications by accepting invitations to review manuscripts (an example of a type of activity that is pandemic-compatible). Perhaps it is a good time for you to widen your public outreach and write for public media? Again (see Rule 1), to fully utilize the potential of social media to disseminate useful information to the general public and reinforce #StayAtHome. You can also get involved with webinars [4] or online conferences [5], either as a participant or organizer. For bioinformaticians, you will be familiar with the online sharing of computational analyses [6] or software tools [7]; for others, this will be a key time to develop skills on multisite collaborations [8]. You can even seek help from online scientific communities [9]. But also keep in mind Rule 1: All you contribute can be undone by overstating your case at a time when exactly what we know and don’t know is made abundantly clear.

## Rule 7: It could be a time for long-cherished goals

As a researcher, there are likely some things you simply cannot do right now. On the other hand, there may be time for either scholarly or professional pursuits that you’ve always had on your To Do list but for which you never had time. There is always that review or paper to be written that is constantly on the back burner, the exploration of a new area of research with never the time to read the background literature, that software that needs to be written or rewritten, a grant you have always meant to write and so on. It is never too late for experimental biologists to sharpen programming skills [10]. Refer to Rule 6 in thinking about these opportunities in terms of what can contribute scientifically in this time of need. An example would be online materials for a broad audience that imparts your knowledge and skill for the common good. For us, this article embraces Rules 6 and 7.

## Rule 8: Reassess your work–family balance

Personal adversity changes everything, including family relationships. When the adversity is population-wide, as in the case of a pandemic, the potential for positive collective impacts at the societal level is simply unparalleled, almost by definition. Conducting research can be an all-consuming challenge to work–life balance. Confinement provides time to reflect and begin to act if you feel your balance may be off. Don’t just use time saved to work, but use it to make a difference to others in nonscientific ways. For many, this will come out of necessity when the daycare center is closed, or aging parents need help. Make those who need you in ways they did not before a priority. Evaluate priorities (see Rule 10) and share the responsibilities fairly and equitably. Reassessment of work–family balance is especially hard with both parents working and your children at home with no childcare. Likewise, if you are on a tenure clock, there is added pressure to perform in impossible circumstances. Look to what your institution offers in help, and discuss the situation with those to whom you report.

## Rule 9: Explore other interests

Exploring interests not related to your research is important at the best of times and especially important at the worst of times. It is all too easy to wander over to the computer and just keep working. Embrace distractions. Think about your physical and mental wellbeing. Other interests are critical here. Is there a way those interests can benefit others less fortunate in this time of need? Perhaps do something that works on emotions in need of bolstering right now. Take humor (https://slydor.com/comics-explained-working-from-home-vs-office/). Perhaps tap into your creativity to create your own comics [11]. Do what works for you that explores your artistic (or really any nonscientific) side. This is also a unique opportunity for researchers with busy schedules that are often fragmented with meetings and such to improve our sedentary lifestyles by getting outside to exercise, hike in nature, and so on (minding the 2-meter criteria interpersonal distances, of course).

## Rule 10: Reevaluate what is important

Suddenly, what used to feel so important is less so in the face of a crisis. Think about what is indeed important now, and consider what you will do differently when it is over. Considering also (like a New Year’s resolution), you may not keep to what you commit to. Writing down what is important during this stressful time and referring back to it in both the near and distant future is, at least, a start. Doing so has nothing to do with doing the research; but, as a researcher, part of that evaluation should be considering the value of the various aspects of your research. Perhaps in the light of recent events, other avenues of inquiry that use your skillset are more or less important than you would have imagined before the pandemic?

Inspiration for this article came from HHG, who provided a first draft, and PEB while (virtually) staring into the eyes of the graduate students that make up his School of Data Science. What can we do next to help? What can you do? Tell us by commenting on this article, using social media, or writing to us directly.
